# Development of Strong and Tough β-TCP/PCL Composite Scaffolds with Interconnected Porosity by Digital Light Processing and Partial Infiltration

**DOI:** 10.3390/ma16030947

**Published:** 2023-01-19

**Authors:** Yanlong Wu, Ruomeng Chen, Xu Chen, Yongqiang Yang, Jian Qiao, Yaxiong Liu

**Affiliations:** 1 School of Mechatronic Engineering and Automation, Foshan University, Foshan 528000, China; 2 Ji Hua Laboratory, Foshan 528200, China; 3 School of Mechanical and Automotive Engineering, South China University of Technology, Guangzhou 510640, China; 4 Key Lab of Intelligent Equipment Digital Design and Process Simulation, Tangshan College, Tangshan 063000, China

**Keywords:** porous composite scaffolds, mechanical properties, digital light processing, infiltration, gradient structure

## Abstract

Strong and tough β-TCP/PCL composite scaffolds with interconnected porosity were developed by combining digital light processing and vacuum infiltration. The composite scaffolds were comprised of pure β-TCP, β-TCP matrix composite and PCL matrix composite. The porous β-TCP/PCL composite scaffolds showed remarkable mechanical advantages compared with ceramic scaffolds with the same macroscopic pore structure (dense scaffolds). The composite scaffolds exhibited a significant increase in strain energy density and fracture energy density, though with similar compressive and flexural strengths. Moreover, the composite scaffolds had a much higher Weibull modulus and longer fatigue life than the dense scaffolds. It was revealed that the composite scaffolds with interconnected porosity possess comprehensive mechanical properties (high strength, excellent toughness, significant reliability and fatigue resistance), which suggests that they could replace the pure ceramic scaffolds for degradable bone substitutes, especially in complex stress environments.

## 1. Introduction

Bioactive bone substitutes, which could be gradually resorbed in vivo and replaced by new bone tissue, thus realizing the self-repair of bone defects, have received increased attention in recent years [1,2,3]. When load-bearing bones or large segmental bones are targeted, it remains a scientific challenge for the scaffolds to maintain both the required interconnected three-dimensional structure and comprehensive mechanical properties (strength and toughness). First, the interconnected macroscopic pore with a range of 100–500 μm are necessary not only for promoting bone ingrowth and vascularization, but also for the transport of nutrients and discharge of metabolic waste [4,5,6]. Second, the bone substitutes need to be strong (sufficient strength) to provide temporary support and transfer appropriate mechanical stimulation to promote new bone regeneration and reconstruction [7,8]. More importantly, toughness is also essential to the bone substitutes, which could increase the reliability and fatigue resistance of the implants to avoid the sudden fracture and maintain mechanical integrity and stability during degradation [9,10].

Biodegradable ceramics, especially β-tricalcium phosphate (β-TCP), are attractive as bone substitutes due to their excellent strength, biocompatibility, degradation, bioactivity and osteoconductivity [8,11,12,13]. Unfortunately, three-dimensional ceramic scaffolds are prone to sudden and catastrophic failure due to their inherent brittleness and low fracture toughness, which limits their application in load-bearing or large segmental bone application. Polymers (e.g., polylactic acid, polylactic-co-glycolic acid, polycaprolactone and gelatin) with excellent toughness have been introduced into ceramic scaffolds as secondary phase material to overcome their inherent brittleness. The fabrication of ceramic/polymer composite structures is a possible approach for not only improving the fragility of ceramics but also regulating their strength and modulus. The most commonly used method for developing ceramic/polymer composites is coating the external surface of a ceramic scaffold with a polymer, which is an effective solution for improving ceramic scaffolds and preserving the interconnected macroscopic pore of the original 3D scaffolds [14,15,16,17]. However, the polymer layer may reduce the biological performance of the ceramic scaffolds and even change their absorbability because the polymer layer prevents calcium, phosphate and other appropriate ions from coming into direct contact with cells and body fluids [14,18,19]. To overcome this problem, full polymer infiltration, a process in which a polymer is introduced into a porous ceramic structure to form a co-continuous phase composite instead of a polymer coating, is receiving increasing attention [10,20,21,22,23]. Indeed, co-continuous phase ceramic/polymer composites show not only a remarkable increase in strength, but also a toughness orders of magnitude higher than that of bare ceramic scaffolds due to the interconnectivity of the 3D polymeric phase interpenetrating structure. Nevertheless, the composite scaffolds produced by the full polymer impregnation method lacks the open macroporous structure, which plays a vital role in promoting cell proliferation, bone growth, and vascularization [4,24]. Recently, some scholars have fabricated ceramic/polymer composite scaffolds with the appropriate porous structures through a suction process, in which a polymer was used to partially fill the macropore structure by applying a vacuum on one end of ceramic scaffolds to create suction [25,26,27]. Although the toughness of the composite scaffolds was impressively improved, the strength of the scaffolds was significantly sacrificed.

The bone substitutes are under a complex mechanical environment when load-bearing bones or large segmental bones are targeted. Thus, the comprehensive mechanical properties of ceramic/polymer composite scaffolds also should be investigated, which is of significance for resorbable bone substitutes. To the best of our knowledge, the mechanical characterization and enhancement of these ceramic/polymer composite scaffolds under quasi-static loading are frequently reported in other studies [21,27,28], but little research has been devoted to their behavior under dynamic loading. Moreover, limited of the current study has been paid to the reliability of ceramic/polymer composite scaffolds with interconnected macroscopic porosity.

Thus, this work proposes a method for fabricating β-TCP/PCL composite scaffolds with not only comprehensive mechanical properties but also suitable macropore structures by combining digital light processing (DLP) technology with a partial infiltration process. First, β-TCP scaffolds (hollow scaffolds) designed with an unconnected and independent double-channel pore structure were fabricated by DLP. Then, one of the channels in the ceramic scaffolds was used for PCL matrix composite impregnation. The PCL matrix composite is the material proposed in this study, which incorporates β-TCP particles into PCL to achieve excellent bioactivity and mechanical properties. For comparison, ceramic scaffolds with the same macropore structure as the composite scaffolds (dense scaffolds) were also manufactured by DLP. Then, the comprehensive mechanical performance of these scaffolds was investigated under both quasi-static (compression and three-point bending) and dynamic (compression) loading conditions. Moreover, the reliability of the composite scaffolds, which is important when the scaffolds are used in complex stress environments, was assessed by compression tests and Weibull statistics.

## 2. Materials and Methods

### 2.1. Raw Materials

Submicron β-TCP ceramic powder (3.07 g/cm^3^, d50 = 0.93 µm, Nanjing Emperor Nano Material, China) and commercial pellet PCL (Molecular weight: 60000, ESUN, Hubei, China) were used as the raw materials. Two types of photopolymer resins, trimethylolpropane triacrylate (TMPTA) and hexanediol diacrylate (HDDA), were purchased from Curease Chemical, China. γ-Aminopropyl triethoxy silane (KH-550, Curease Chemical, China) and bis-2,4,6-trimethylbenzoyl-phenyl phosphine oxide (819, Guanzhou Card Finn Biological Technology, China) were chosen as the dispersant and photoinitiator, respectively, in this study. Graphite (Shanghai Naiou Nano Technology Co., Ltd., Shanghai, China) with a mean particle size of d50 = 2.1 μm was employed to inhibit the light scattering phenomenon in ceramic suspensions. N,N dimethyl acetamide (Shanghai Aladdin Bio-Chem Technology Co., Ltd., Shanghai, China) was used as the PCL solvent.

### 2.2. Fabrication of β-TCP Scaffolds

**Figure 1:**
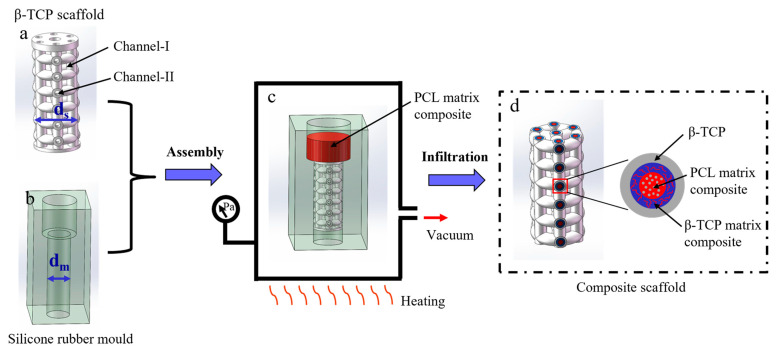
Schematic illustrations of ceramic/polymer composite scaffolds with gradient structure: (**a**) ceramic scaffolds fabricated by DLP, (**b**) silicon rubber mold fabricated by mold method, (**c**) followed by PCL-impregnation of the sintered ceramic scaffolds, (**d**) ceramic/polymer composite scaffolds by cutting off both ends.

As shown in Figure 1a, the β-TCP scaffolds for impregnation had an unconnected and independent double-channel pore structure, where one of the channels was used for bone ingrowth and vascularization (channel I) and the other was used for polymer impregnation (channel II). The scaffolds had a total tailored porosity of 62.8%, and the porosity and tailored pore size for channel II were 25.1% and 500 μm, respectively. Furthermore, the scaffold was designed such that one end of the scaffold face is completely closed and the other only keeps channel II for polymer impregnation connected to the outside. The method used to prepare the β-TCP ceramic suspension was described in our previous article [29]. Briefly, a resin-based β-TCP ceramic suspension with a solid loading of 40% was prepared by ball mixing β-TCP powder, two resin monomers (HDDA:TMPTA = 1:1), dispersant KH-550, photoinitiator 819 and graphite (6 wt‰, compared with β-TCP ceramic powder). After ball milling, the suspension was poured into a printing chamber, and β-TCP scaffolds with both channel I and channel II (hollow scaffold, see Figure 1a) for impregnation and β-TCP scaffolds with only channel I (dense scaffold, see Figure 6c,d) for comparison were fabricated by a DLP printer (M-Jewelry U30, MakeX Ltd., Ningbo, China). After printing, these scaffolds were heat-treated, first at 600 °C for 2 h to remove the polymer network and then at 1100 °C for 2 h in an air atmosphere.

### 2.3. PCL Matrix Composite Preparation

It has been suggested that doping β-TCP particles into the PCL matrix improves the biological and mechanical properties of PCL, especially the elasticity modulus [30]. To ensure that the ceramic particles were uniformly dispersed in the PCL matrix, first, PCL was dissolved in N,N-dimethyl acetamide in a 1:2 w/v ratio with a water bath at 80 °C for 60 min. Then, the β-TCP ceramic particles (20 wt%, according to PCL) were added to the PCL solution. The mixed solution was stirred for 60 min and dispersed in an ultrasound cleaner for 15 min. Finally, the mixed solution was rinsed with deionized water several times and dried in a vacuum environment at 200 °C for 1 h.

### 2.4. PCL Matrix Composite Infiltration Process

In this work, we used a vacuum infiltration method with easily removable silicone rubber molds to fabricate composite ceramic/polymer scaffolds. A high vacuum was applied to force the PCL melt to fill channel II of the β-TCP scaffolds. Removable silicone rubber molds were introduced to obtain net-shaped composite ceramic/polymer scaffolds with gradient structures. As shown in Figure 1, a sintered β-TCP scaffold was assembled with silicone rubber molds for the filling of the PCL melt (as seen in Figure 1c). The cavity diameter of the silicone rubber mold was 0.8–0.9 times the diameter of the sintered β-TCP scaffolds (as seen in Figure 1a,b). Then, the PCL matrix composite was placed in a silicone rubber mold at 200 °C in a vacuum drying oven. When the PCL matrix composite was melted, the scaffolds were kept at high vacuum environment for 60 min, 120 min, 180 min, 240 min and 300 min. Subsequently, the scaffolds were cooled to room temperature by removing them from the vacuum drying oven, and both ends of the scaffolds were cut off.

### 2.5. Microstructure and Mechanical Property Characterization

The morphologies and pore dimensions of the hollow scaffolds, dense scaffolds and composite scaffolds infiltrated for different times (60 min, 120 min, 180 min, 240 min and 300 min) were analyzed by using scanning electron microscopy (SEM, SU-8010 Hitachi Ltd., Tokyo, Japan) on the metallized samples. The thickness of the PCL penetration into the ceramic matrix was also evaluated.

The mechanical responses of the hollow, dense and composite scaffolds (infiltrated for 240 min) were evaluated under compression and three-point bending tests. Five samples in each condition were placed in air using a universal testing machine (CMT4304, Sans Ltd., Shanghai, China) with a constant crosshead speed of 0.5 mm/min. The force was applied in a direction perpendicular to the printing plane. The dimensions of the samples were approximately φ4 × 8 mm and 21.5 × 5.7 × 3.4 mm^3^ for the uniaxial compressive and three-point bending tests, respectively. Stress–strain curves were calculated through the normalization of the captured load versus displacement data using the initial external dimensions of each sample. The compressive strength (σ_c_) and flexural strength (σ_f_) were evaluated by the maximum stress values obtained from the nominal stress–strain curves. The toughness of the compressed samples was estimated as the strain energy density for three values: the strain at the maximum compressive strength (G_max_), at 10% strain (G_0.1_) and at 20% strain (G_0.2_) [20], obtained from the corresponding integrals of the nominal stress–strain curves. Similarly, the toughness of the three-point bending samples was estimated as the fracture energy density for two values: the strain at the maximum compressive strength (G_max_) and at 5% strain (G_0.05_) [9].

To evaluate the reliability of these samples, another fifteen samples (hollow scaffolds, dense scaffolds and composite scaffolds infiltrated for 240 min) were tested under uniaxial compression. Strength data were analyzed using Weibull statistics, where the failure probability, *P*, was given by [21,22]

(1) $$P=1-\exp\left[-{\left(\sigma /\sigma_{0}\right)}^{m}\right]$$

where *m* is the Weibull modulus, which is a measure of the sample reliability, and *σ*_0_ is the central value of the strength distribution. All data are expressed as the means with the standard deviations as the errors.

To compare the resistance to the dynamic fracture for the dense scaffolds and the composite scaffolds infiltrated for 240 min, three samples of each scaffold were tested under compression with cyclic stress. The maximum cyclic stress was half of the average compressive strength of each scaffold. The stress ratio and loading cycle were set to 0.1 and 3.5 s, respectively. The displacements of the loading points and the number of cycles were recorded and analyzed.

All values are expressed as the mean ± standard error of the mean and were analyzed. All experiments were repeated at least three times. Differences between mean values of normally distributed data were evaluated by Tukey’s post hoc test. Finally, 0.01< *p* < 0.05 was considered to indicate a statistically difference between groups, and *p* < 0.01 was considered to indicate a statistically significant difference between groups.

## 3. Results

### 3.1. Microstructure of the Porous β-TCP/PCL Composite Scaffolds

**Figure 2:**
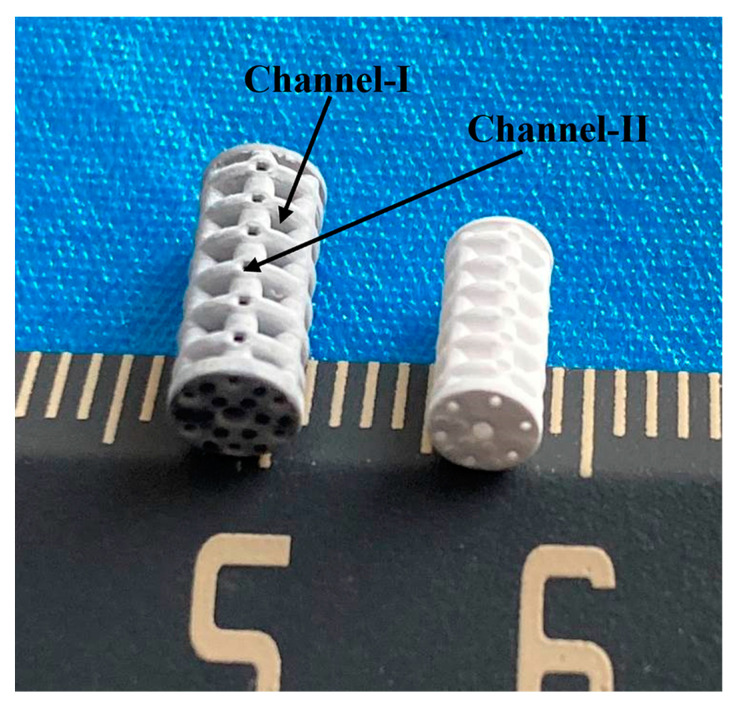
Photographs of β-TCP ceramic scaffolds (hollow scaffolds) fabricated for impregnation by DLP process (**left**: green scaffolds, **right**: sintered scaffolds).

Figure 2 shows photographs of the green (left) and sintered (right) β-TCP ceramic scaffolds (hollow scaffolds) fabricated by the DLP process for impregnation. After sintering, the resin and other organic materials were removed, and the scaffolds changed from grey to white. An independent double-channel pore structure was clearly observed for these samples. The channel II average pore size of the green scaffolds was 445.8 ± 16.5 μm, which was smaller than the designed pore size (500 μm). This is mainly due to light scattering on the photopolymerizable ceramic suspensions during the manufacturing process. After sintering, these scaffolds showed obvious shrinkage after sintering, and the linear shrinkages of these scaffolds in the XY direction and Z direction were 12.2 ± 0.5% and 14.1 ± 0.6%, respectively.

**Figure 3:**
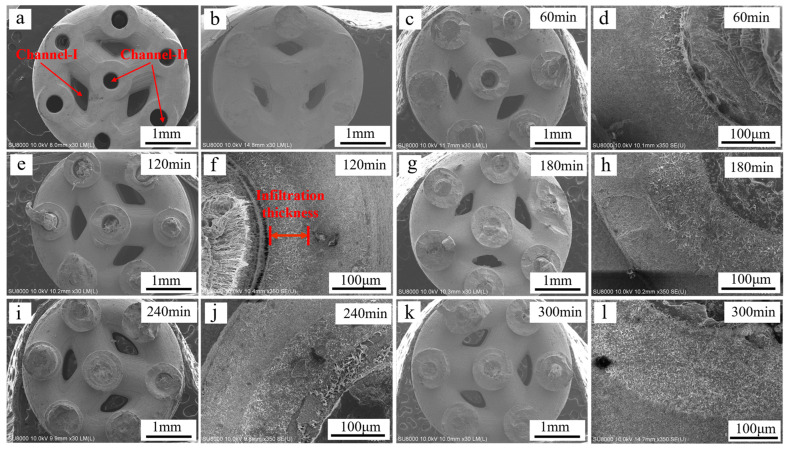
SEM micrograph of scaffolds cross-sections for (**a**) hollow scaffolds (**b**) dense scaffolds and composite scaffolds infiltrated with the PCL matrix composite for different infiltration times: (**c**,**d**) 60 min, (**e**,**f**) 120 min, (**g**,**h**) 180 min, (**i**,**j**) 240 min, (**k**,**l**) 300 min.

**Figure 4:**
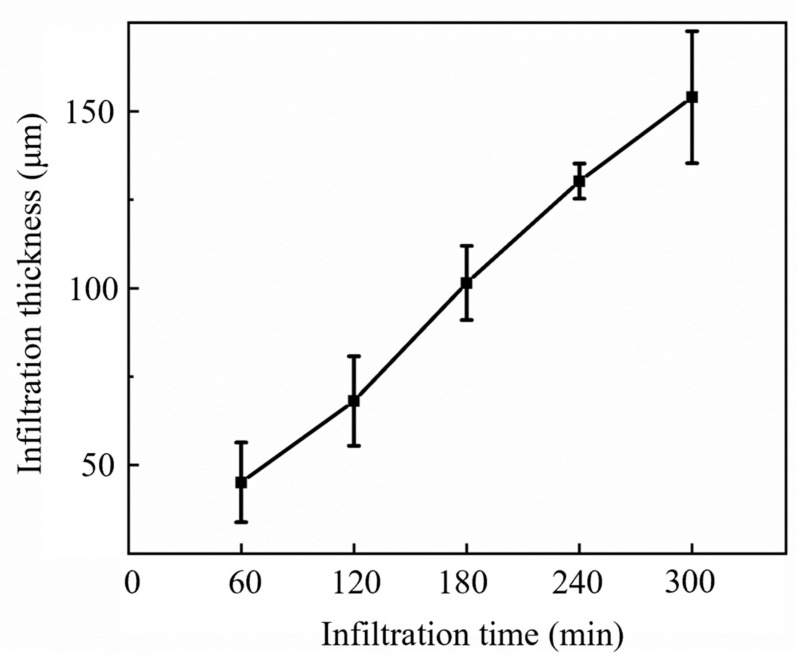
The infiltration thickness of composite scaffolds with infiltration time.

Figure 3a–l show SEM micrographs of the cross-sections of the hollow scaffolds, dense scaffolds and composite scaffolds infiltrated with the PCL matrix composite for different infiltration times. The images show that the hollow and dense scaffolds maintained a good macroscopic pore structure, and the channel II average pore size of the sintered hollow scaffolds was 391.3 ± 19.7 μm (Figure 3b), with a 12.2 ± 0.8% measured shrinkage, which is close to the overall shrinkage of the hollow scaffolds. After immersing the hollow scaffolds in the PCL matrix composite, a gradient structure was formed (Figure 3c–l). It is obvious that the PCL matrix composite not only completely filled channel II but also partly infiltrated the open micropores in the bioceramic. It is worth noting that channel I still preserved a good predesigned structure, and the PCL matrix composite did not penetrate it, which is necessary for cell proliferation, bone growth, and vascularization. We inferred that the molten PCL matrix composite first infiltrated channel II of the scaffold, and after channel II was full of the molten PCL matrix composite, it infiltrated the open micropores along the inner walls of the channel. As shown in Figure 4, the infiltration thickness, namely, the penetration distance of the PCL matrix composite into the pure ceramic, increased linearly with infiltration time. The infiltration thickness in the pure PCL gradient structure was reported as approximately 150 μm with a 60 min immersion time in previous work [10], while that of the PCL matrix composite was less than 50 μm. This should be attributed to the fact that the addition of ceramic particles may reduce the thermal fluidity of PCL, which is not conducive to the penetration of PCL into the ceramic microstructure.

### 3.2. Microstructure of the Gradient Structure

**Figure 5:**
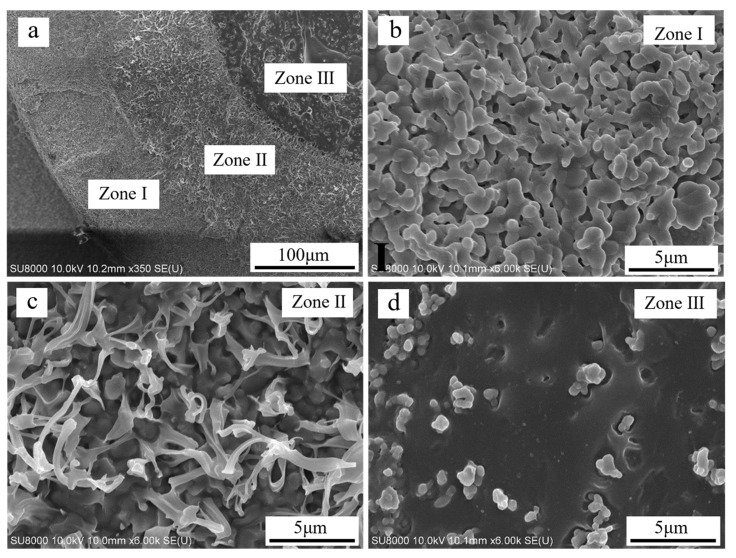
SEM micrograph of (**a**) gradient structure of β-TCP/PCL composite scaffolds: (**b**) β-TCP ceramic, (**c**) β-TCP matrix composite, (**d**) PCL matrix composite.

As shown in Figure 5a, the gradient structure of the porous β-TCP/PCL composite scaffolds (infiltrated for 180 min) comprised pure β-TCP (Figure 5b), the β-TCP matrix composite (Figure 5c) and the PCL matrix composite, marked as zone I, zone II and zone III, respectively. The microstructure of zone I, where infiltration did not occur, with a thickness of 106.9 ± 11.2 μm contained abundant interconnected micropores less than 1 μm. These micropores contributed to the infiltration of the PCL matrix composite into the ceramic and resulted in a gradient structure. The formation of the zone Ⅱ structure with a thickness of 130.5 ± 4.9 μm was caused by the penetration of PCL into the ceramic micropores under high-vacuum and high-temperature conditions, which is consistent with the phenomenon that solutions (e.g., sodium silicate, ethyl cyanoacrylate) were impregnated into other solid materials (e.g., wood, green ceramics) in a vacuum environment [31,32]. Therefore, we inferred that the penetration of PCL into the microstructure of the ceramic matrix in this study was mainly due to the combined effect of wetting, capillary and adsorption mechanisms. It is worth noting that a large number of PCL microfibers grew from the ceramic micropores in zone Ⅱ, which may have been helpful for preventing the growth of microdefects on the ceramic. As shown in Figure 5d, it is evident that a number of ceramic particles were dispersed in the PCL matrix. However, some ceramic particles were still agglomerated.

### 3.3. Mechanical Properties of the Porous β-TCP/PCL Composite Scaffolds

**Figure 6:**
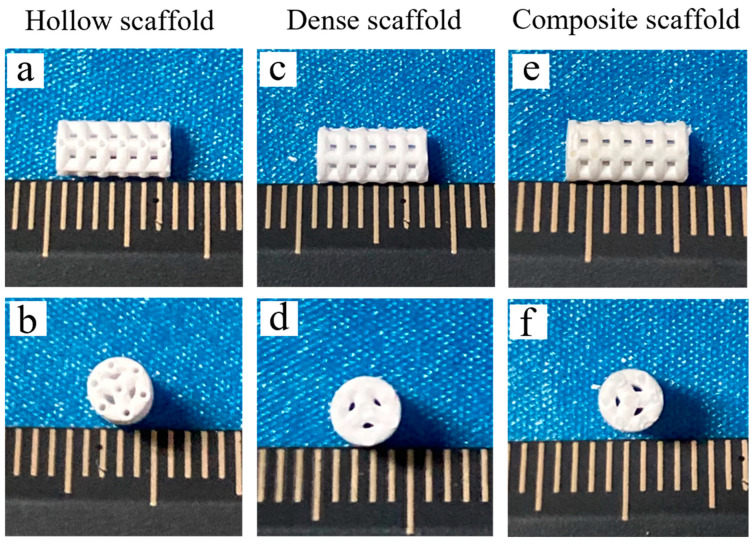
Photographs of β-TCP ceramic scaffolds fabricated by the DLP process: (**a**,**b**) hollow scaffolds, (**c**,**d**) dense scaffolds, (**e**,**f**) composite scaffolds fabricated by the infiltration process.

**Figure 7:**
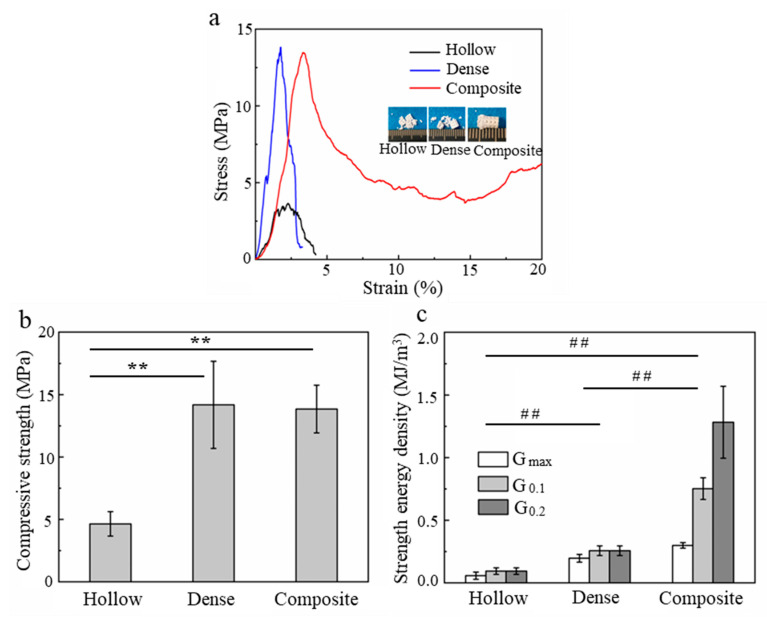
(**a**) Representative uniaxial compressive stress-strain curves (**b**) compressive strength (**c**) strain energy density for the different scaffolds. (Data presented are the mean ± standard error of the mean, n = 5, ** ## *p* < 0.01).

As shown in Figure 6, the scaffolds (hollow scaffolds, dense scaffolds and composite scaffolds infiltrated for 240 min) were fabricated by DLP or infiltration processes for uniaxial compressive experiments. Figure 7a shows the representative compressive stress–strain curves corresponding to each of them, showing significantly different responses. The hollow and dense scaffolds failed at the maximum stress and then soon suffered a complete collapse with the load dropping to zero, which is a typical brittle fracture behavior. However, a stress plateau in the stress–strain curves of the composite scaffolds was reached at a large strain after fracture occurred. It is interesting that when the strain reached 20%, the composite scaffolds still maintained a significant residual load-bearing capacity (over 5 MPa). Furthermore, it was found that the hollow and dense scaffolds crumbled in many parts, whereas the composite scaffolds retained their shape and did not crumble after the compression test, even with a strain over 20%. It was inferred that the composite scaffolds maintained their integrity under a large strain due to macro- and micro-PCL.

Figure 7b summarizes the compressive strengths of the hollow, dense and composite scaffolds. The compressive strength of the composite scaffolds was significantly increased compared to that of the hollow scaffolds—up to 13.8 ± 1.9 MPa from 4.6 ± 0.1 MPa. This may be responsible for two strengthening mechanisms that were revealed in other studies [33]: stress shielding, in which the PCL filled the macropores (channel II) and could transfer and bear part of the stress; and defect healing, in which the PCL microfibers healed the defects in the hollow ceramic scaffolds. It was obvious that the compressive strength of the composite scaffolds was very close to that of the dense scaffolds (14.2 ± 3.4 MPa), although they have identical macroscopic pore structures. This is in contrast with other results on composite scaffolds fabricated by DLP or DIW, where the compressive strength of the composite scaffolds only reached one-third of the strength of dense scaffolds [17,19]. This can be explained by the controllable gradient structures fabricated playing a significant role in enhancing the compressive strength of the composite scaffold, where the PCL microfibers (see Figure 5d) enhance the union between the organic and inorganic phases and provide an attractive combination of strength. Moreover, the PCL matrix composite used in this work, instead of pure PCL, has a higher modulus, which may allow greater stress to be endured.

Regarding the quantification of the toughness of the composite scaffolds, Figure 7c shows the strain energy density of the hollow, dense and composite scaffolds at three strain values: the strain at the maximum stress (G_max_), at 10% strain (G_0.1_) and at 20% strain (G_0.2_). There was an obvious reduction (over 60%) in the strain energy density of the β-TCP scaffolds when the dense rods were locally substituted by hollow struts. However, after PCL matrix composite impregnation, the strain energy density (G_0.1_, G_0.2_) of the composite scaffolds was significantly improved by more than 800% compared to that of the hollow scaffolds. Moreover, despite having a similar compressive strength and the same macroporosity, the strain energy density G_max_, G_0.1_ and G_0.2_ of the composite scaffolds were 1.5, 2.9 and 4.9 times that of the dense scaffolds, respectively. The toughening achieved may be due to a crack bridging mechanism facilitated by the microfibrils (as shown in Figure 5c) and macrofibrils (as shown in Figure 5d) that bridge the cracks and even change the direction of the cracks as they propagate.

**Figure 8:**
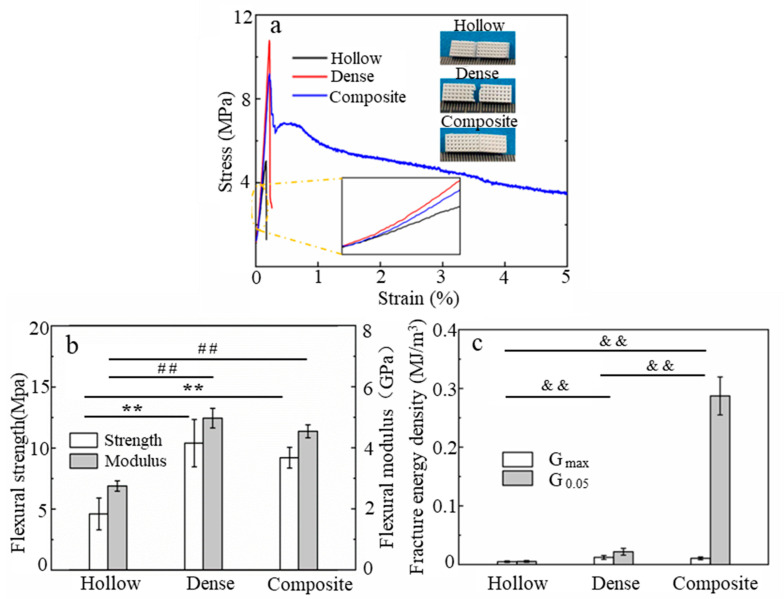
(**a**) Representative three-point bending stress-strain curves (**b**) flexural strength (**c**) fracture energy density for the different scaffolds. (Data presented are the mean ± standard error of the mean, n = 5, ** ## && *p* < 0.01).

The changes in the fracture behaviors were also clearly evidenced when the composite scaffolds were subjected to bending stress and compared to the dense and hollow scaffolds, as shown in Figure 8a. While a linear elastic behavior followed by a stress drop associated with sudden and catastrophic fracture was observed for both the hollow and dense scaffolds, the composite scaffolds initially exhibited the same trend, followed by a gradual decline after reaching the maximum stress and then retaining a significant load-bearing capacity even at 5% strain. According to the curve, the two kinds of ceramic scaffolds completely broke into two parts after the three-point bending tests, but the composites stretched and bridged the fractured skeleton together at relatively high strains (over 5%) due to the gradient structure and the plastic deformation of the PCL phase. Such a behavior was also observed for metal/polymer composites [34]. 

The flexural strength and flexural modulus results estimated from these curves are summarized in Figure 8b. As indicated, the flexural strength of the composites (9.2 ± 0.9 MPa) was strongly improved compared with that of the hollow scaffolds (4.6 ± 4.6 MPa). However, there were no substantial flexural strength differences between the dense (10.4 ± 1.9 MPa) and composite scaffolds in terms of bending. Similar to the flexural strength, the bending modulus of the composite was only 9.5% higher than that of the dense scaffolds but was 1.81 times that of the hollow scaffolds. These results were obtained because although the PCL microfibers in the gradient structure enhanced the strength and modulus of the composite scaffolds, the PCL in the macropores (channel II) instead of the dense ceramic was not conducive to the strength and modulus of the composite scaffolds, and the latter effect was slightly more significant.

The toughness of these scaffolds in terms of bending, evaluated as fracture energy densities (G_max_ and G_0.05_), is shown in Figure 8c. The composite scaffolds showed a fracture energy density at 5% strain (G_0.05_) more than one order of magnitude higher than that of the other scaffolds: 0.287 ± 0.032 MJ/m^3^ vs. 0.023 ± 0.006 MJ/m^3^ and 0.005 ± 0.002 MJ/m^3^ for the dense and hollow scaffolds, respectively. The achieved toughness improvement was clearly superior to that observed for compression.

**Figure 9:**
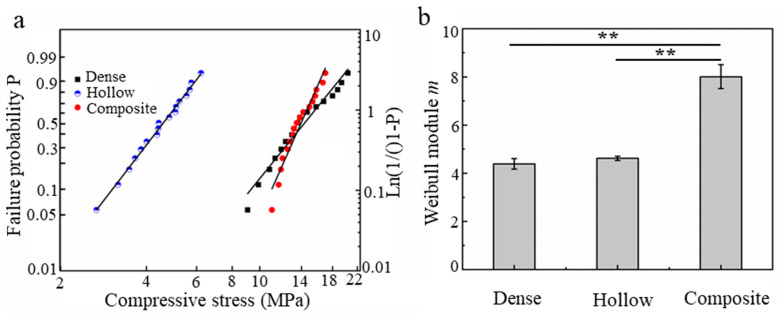
(**a**) Weibull plot of the compressive strength data for the different scaffolds, straight lines are best fits to data using the Weibull probability function (**b**) Weibull modulus, m, for the different scaffolds. (Data presented are the mean ± standard error of the mean, n = 15, ** *p* < 0.01).

As shown in Figure 9, the Weibull modulus of the composite scaffolds reached 8.01 ± 0.49, which is over 1.7 times that of both the dense (m = 4.39 ± 0.22) and hollow (4.62 ± 0.09) scaffolds. The Weibull modulus of the composite scaffolds was equal to or superior to a previously reported value for human cortical bone (m = 8) [21]. Indeed, the observed increase in the Weibull modulus might be associated with the fact that the PCL microfiber, as the microporous defect healing agent, can inhibit ceramic crack propagation and that the PCL matrix composite filled in channel II endows the composite scaffolds with a remarkable toughness. These results indicate that the stability and reliability were significantly improved after PCL impregnation. In addition, the Weibull modulus, which depended on the inherent properties of the β-TCP ceramic and manufacturing process, of the dense and hollow scaffolds were not significantly different.

**Figure 10:**
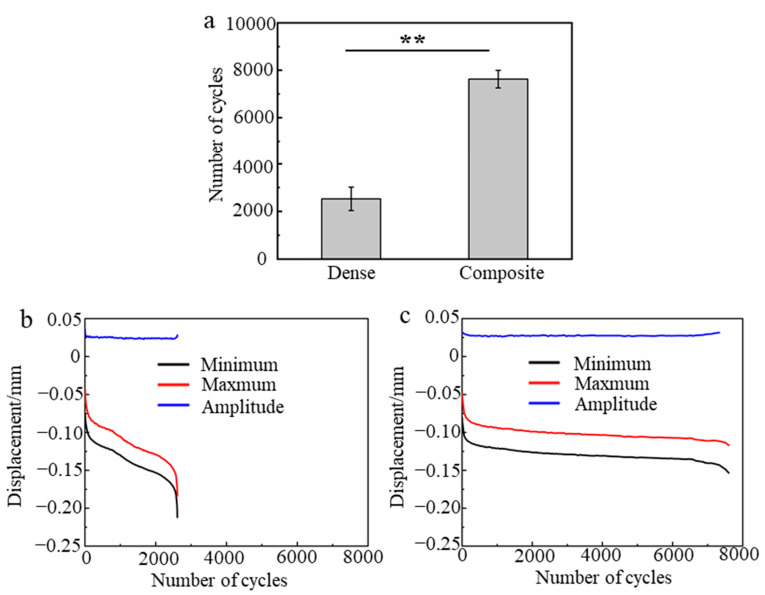
Compressive fatigue performances of the dense and composite scaffolds. (**a**) Number of cycles for dense and composite scaffolds, representative evolutions of the minimum, maximum and amplitude of the displacement: (**b**) dense scaffolds (**c**) composite scaffolds. (Data presented are the mean ± standard error of the mean, n = 3, ** *p* < 0.01).

The dynamic fracture resistance of the dense scaffolds and the composite scaffolds were investigated and compared, as shown in Figure 10. The fatigue life (number of cycles) of the composite was 3.0 times that of the dense scaffolds (Figure 10a). This indicates that the composite scaffolds may be more suitable for a complex stress environment when they are used as a bone substitute. The displacement amplitude of the composite scaffolds was higher than that of the dense scaffolds (Figure 10b,c). This implies that the dynamic modulus of the composites was lower than that of the dense scaffolds, which is in accordance with the results of the three-point bending tests. It is evident that the minimum and maximum displacements rapidly decreased with the number of cycles for the dense scaffolds (Figure 10b), indicating the continuous fracture of local struts. In contrast, the minimum and maximum displacements of the composite scaffolds decreased slowly with the number of cycles, indicating good resistance to the fracture of local struts. This result confirms that the PCL phase in the ceramic plays an important role in resisting the cracking of the composite scaffolds.

## 4. Conclusions

Porous β-TCP/PCL composite scaffolds with gradient structures, which showed outstanding mechanical performance (high strength, excellent toughness, significant reliability and fatigue resistance), were successfully fabricated in this study by combining DLP technology and a partial infiltration process. The main results can be summarized as follows:

1. The porous β-TCP/PCL composite scaffolds produced by a two-step process had a controllable gradient structure and interconnected three-dimensional structure that facilitated bone growth and vascularization.
2. The porous β-TCP/PCL composite scaffolds showed compressive and bending strengths similar to those of ceramic scaffolds with the same macroscopic pore structure (dense scaffolds) but significantly higher compressive and bending strengths than those of unimpregnated ceramic scaffolds (hollow scaffolds). Moreover, the toughness (strain energy density and fracture energy density) of the composite scaffolds was several times or even an order of magnitude higher than that of both dense and hollow scaffolds. The improvement of the composite scaffolds was due to the addition of PCL, which could heal defects and bridge microscopic cracks.
3. The porous β-TCP/PCL composite scaffolds also showed excellent reliability. The Weibull modulus of the composite scaffolds reached 8.01 ± 0.49, which was ~1.7 times higher than that of both dense and hollow scaffolds. The Weibull modulus of the composite scaffolds was comparable to that of natural bone due to polymer reinforcement and toughening.
4. Furthermore, the porous β-TCP/PCL composite scaffolds exhibited better resistance than the dense and hollow scaffolds because the number of cycles of the composite under dynamic loading was 3.0 times that of the dense scaffolds.

In conclusion, porous β-TCP/PCL composite scaffolds are expected to replace pure ceramic scaffolds in bone tissue engineering to achieve a greater range of applications, especially in complex stress environments. However, the composite scaffolds lack structural optimization, especially the distribution of PCL and ceramic materials in the scaffolds, which may endow these scaffolds with better mechanical properties. Furthermore, it is important to evaluate the degradation and dynamic mechanical properties of composite scaffolds with the time of implantation. In future work, the improvements in the mechanical properties of the composite scaffolds by optimizing their structure and material distribution, as well as studying their degradation kinetics in vivo and in vitro will be further explored.

## Data Availability

Not applicable.
